# Household income and maternal education in early childhood and risk of overweight and obesity in late childhood: Findings from seven birth cohort studies in six high-income countries

**DOI:** 10.1038/s41366-022-01171-7

**Published:** 2022-07-11

**Authors:** Pär Andersson White, Yara Abu Awad, Lise Gauvin, Nicholas James Spencer, Jennifer J. McGrath, Susan A. Clifford, Béatrice Nikiema, Junwen Yang-Huang, Jeremy D. Goldhaber-Fiebert, Wolfgang Markham, Fiona K. Mensah, Amy van Grieken, Hein Raat, V. W. V. Jaddoe, Johnny Ludvigsson, Tomas Faresjö, EPOCH Collaborative Group, Jennifer J. McGrath, Louise Séguin, Nicholas J. Spencer, Kate Pickett, Hein Raat, Yara Abu Awad, Pär Andersson White, Guannan Bai, Philippa Bird, Susan A. Clifford, Åshild Faresjö, Tomas Faresjö, Kate L. Francis, Lise Gauvin, Sharon Goldfeld, Jeremy D. Goldhaber-Fiebert, Johnny Ludvigsson, Wolfgang Markham, Fiona K. Mensah, Béatrice Nikiéma, Elodie O’Connor, Sue Woolfenden, Junwen Yang-Huang

**Affiliations:** 1 Department of Health, Medicine and Care, General Practice, Linköping University, SE-58183 Linköping, Sweden; 2 Crown Princess Victoria Children´s Hospital, Region Östergötland, SE-58185 Linköping, Sweden; 3 PERFORM Centre, Concordia University, H4B 1R6 Montreal, QC Canada; 4 Centre de recherche du Centre Hospitalier de l’Université de Montréal, H2X 0A9 Montréal, QC Canada; 5 École de santé publique, Université de Montréal, H2X 0A9 Montréal, QC Canada; 6 Division of Health Sciences, Warwick Medical School, University of Warwick, CV4 7AL Coventry, UK; 7 Murdoch Children’s Research Institute, Melbourne, VIC 3052 Australia; 8 Department of Paediatrics, The University of Melbourne, Melbourne, VIC 3052 Australia; 9 Cree Board of Health and Social Services of James Bay, Department of Program Development and Support, G0W 1C0 Chisasibi, QC Canada; 10 The Generation R Study Group, Erasmus University Medical Center, 3000 CA Rotterdam, the Netherlands; 11 Department of Public Health, Erasmus University Medical Center, 3000 CA Rotterdam, the Netherlands; 12 Stanford University, 94305 Stanford, CA USA; 13 Department of Pediatrics, Erasmus University Medical Center, 3000 CA Rotterdam, the Netherlands; 14 Department of Epidemiology, Erasmus University Medical Center, 3000 CA Rotterdam, the Netherlands; 15 Division of Pediatrics, Dept of Biomedical and Clinical Sciences, Linköping University, SE-58185 Linköping, Sweden; 16 PI, Concordia University, Montréal, QC Canada; 17 co-PI, Université de Montréal, Montréal, QC Canada; 18 co-PI, University of Warwick, Coventry, UK; 19 co-PI, University of York, Coventry, UK; 20 co-PI, Erasmus MC, Rotterdam, The Netherlands; 21 Concordia University, Montréal, QC Canada; 22 Crown Princess Victoria Children’s Hospital, Linköping, Sweden; 23 Erasmus MC, Rotterdam, The Netherlands; 24 Bradford Institute for Health Research, Coventry, UK; 25 The University of Melbourne, Melbourne, VIC Australia; 26 Linköping University, Linköping, Sweden; 27 Royal Children’s Hospital, Melbourne, VIC Australia; 28 Centre de recherche du CHUM & Université de Montréal, Montréal, QC Canada; 29 The Royal Children’s Hospital Melbourne, Melbourne, VIC Australia; 30 Stanford University, Stanford, CA USA; 31 University of Warwick, Coventry, UK; 32 Université de Montréal, Montréal, QC Canada; 33 University of New South Wales & Sydney Children’s Hospital, Melbourne, VIC Australia

**Keywords:** Epidemiology, Risk factors, Obesity

## Abstract

**Background/objectives:**

This study analysed the relationship between early childhood socioeconomic status (SES) measured by maternal education and household income and the subsequent development of childhood overweight and obesity.

**Subjects/methods:**

Data from seven population-representative prospective child cohorts in six high-income countries: United Kingdom, Australia, the Netherlands, Canada (one national cohort and one from the province of Quebec), USA, Sweden. Children were included at birth or within the first 2 years of life. Pooled estimates relate to a total of *N* = 26,565 included children. Overweight and obesity were defined using International Obesity Task Force (IOTF) cut-offs and measured in late childhood (8–11 years). Risk ratios (RRs) and pooled risk estimates were adjusted for potential confounders (maternal age, ethnicity, child sex). Slope Indexes of Inequality (SII) were estimated to quantify absolute inequality for maternal education and household income.

**Results:**

Prevalence ranged from 15.0% overweight and 2.4% obese in the Swedish cohort to 37.6% overweight and 15.8% obese in the US cohort. Overall, across cohorts, social gradients were observed for risk of obesity for both low maternal education (pooled RR: 2.99, 95% CI: 2.07, 4.31) and low household income (pooled RR: 2.69, 95% CI: 1.68, 4.30); between-cohort heterogeneity ranged from negligible to moderate (*p*: 0.300 to < 0.001). The association between RRs of obesity by income was lowest in Sweden than in other cohorts.

**Conclusions:**

There was a social gradient by maternal education on the risk of childhood obesity in all included cohorts. The SES associations measured by income were more heterogeneous and differed between Sweden versus the other national cohorts; these findings may be attributable to policy differences, including preschool policies, maternity leave, a ban on advertising to children, and universal free school meals.

## Introduction

The prevalence of overweight and obesity in children and adolescent increased from the 1970s to 2000 in Western Europe and high income English speaking countries. Since then, the levels have plateaued in northern and western Europe, while in the US there is evidence supporting a continuing increase but at a slower pace than during previous decades [1–3]. A majority of studies that focus on the relationship between socioeconomic status (SES) and childhood obesity show that social inequalities, or the SES differences, have continued to increase [4].

The association between SES and adult obesity was established already at the time of the Black Report in the early 1980s [5]. The evidence for an association between low parental SES and overweight/obesity in children is less prominent, but in recent years, most studies in high-income countries show an association with overweight/obesity being more prevalent amongst more socioeconomically disadvantaged families [6, 7]. SES inequalities in child overweight/obesity are increasing in most high-income countries, but differences between countries with similar economic resources are evident. Direct comparisons of gradients between countries are difficult since definitions of SES vary considerably between studies [8]. The use of odds ratios (ORs) instead of risk ratios (RRs) also reduce comparability between studies as ORs change with prevalence [9]. Compared to relative differences, absolute inequality in child overweight/obesity is more important from a public health perspective than relative differences; yet, few studies use absolute measures such as the Slope Index of Inequality (SII) [10].

Potential differences in SES gradients between countries must be viewed in relation to policies in each country/jurisdiction. Ecological studies suggest that countries and states in the USA with high income inequality, measured by the GINI coefficient, also have higher prevalence of childhood overweight [11, 12]. Social/family policies have been associated with decreased child obesity prevalence [13]. For example, physical activity time in schools [14], provision of universal free school meals [15], regulations on advertisement to children [16], and measures for active transport (walking/bicycling) are all associated with decreases in the prevalence of obesity in childhood [17]. Country-level income inequality also merits further investigation, especially the use of prospective data that includes measures of SES in early childhood and its association with overweight/obesity in later childhood.

Our study uses harmonized SES while adjusting for confounding variables, which makes comparison between the included international cohorts more robust. We hypothesize that social gradients in overweight/obesity by household income and maternal education in early childhood will be observed, and the slopes of these gradients will vary according to cohort/country.

### Aim of the study

Using data from seven prospective birth cohorts in six high-income countries, this study aims to analyse the longitudinal relationships in both relative and absolute terms between early childhood SES and the development of overweight and obesity at age 8–11 years.

## Subjects and methods

### Data sources

The Elucidating Pathways of Child Health inequalities (EPOCH) study draws on data from seven prospective birth cohort studies from six high income countries to explore the pathways from early SES exposure to child health outcomes at age 8–11 years. Data were derived from: Sweden, All Babies in Southeast Sweden (ABIS); Rotterdam, The Netherlands, Generation R (GenR); Quebec, Canada, Quebec Longitudinal Study of Child Development (QLSCD); Canada (all provinces), National Longitudinal Study of Children and Youth; Canada (NLSCY); Australia, Longitudinal Study of Australian Children birth cohort (LSAC); The United Kingdom, the Millennium Cohort Study (MCS); and USA, National Longitudinal Study of Children and Youth (USNLSY). Profiles of each study cohort including study design, weighting, and treatment of missing values are provided in Table S1 Cohort profiles (Supplementary data).

Samples from the cohorts were broadly representative of the target populations. In the USNLSY (USA), children born to mothers under the age of 24 years were not enrolled, thus excluding at least 5% of children in this US cohort [18].

Pooled estimates relate to a total of 26,565 included children in this study.

Each cohort enrolled samples of children at birth or within the first 2 years of life. Weights and/or imputation accounting for differential attrition and non-response were applied in all cohorts; five cohorts also applied weights that allowed comparison to their reference populations (QLSCD, NLSCY, LSAC, MCS, USNLSY). The seven cohorts included were intentionally selected as they represent countries across the income inequality gradient as measured by GINI-coefficients; and have differing social/family policies, see Table S2 (Supplementary data).

### Study variables

#### BMI measurement

Height and weight were measured by trained staff in four cohorts (QLSCD, GenR, LSAC, MCS) and were reported by parents in three cohorts (ABIS, NLSCY, USNLSY). These data were collected at age 8–9 years (ABIS, LSAC), age 9–10 years (GenR), or 10–11 years (QLSCD, NLSCY, MCS, USNLSY). Body mass index (BMI) was calculated as BMI = weight(kg)/height(m)^2^ and dichotomized according to the cut-offs for overweight and obese, using the latest International Obesity Task Force age and sex specific cut-offs defined by Cole et al. [19].

#### Measures of socioeconomic status

Household income and maternal education, measured within the first 5 years of life, were available in all cohorts allowing harmonization of these SES variables. Ages at which household income was collected are shown in Table S1 (Supplementary data) which also shows the differences in income date collection (gross income or net of tax). Income data were collected by questionnaire in six out of seven cohorts and by crosslinking with national register in one cohort (ABIS, Sweden). Children were categorized into three groups according to their household income at baseline: high income was defined as an income ≥5th quintile of the original cohort, middle income was defined as 2nd to 4th quintile, and low income was defined as ≤1st quintile.

Maternal education was self-reported via questionnaire at birth or within the first year of life and categorized into three levels according to the International Standard Classification of Education (ISCED): Low education = ISCED I-II, middle education = ISCED III-IV, and high education (university or other higher education) ISCED V-VII.

#### Potential confounders

Confounders were specified a priori using these criteria: associations with both early childhood SES and overweight/obesity that may potentially result in spurious associations between them. Potential confounders included were child’s sex, mother ethnicity, and maternal age at birth, see Table 1.

**Table 1:** Sample characteristics of maternal education, household income, and confounding variables by cohort.

	ABIS Sweden *N* = 3984	QLSCD Quebec *N* = 1334	GenR Netherlands *N* = 7393	NLSCY Canada *N* = 1356	LSAC-B Australia *N* = 4085	MCS UK *N* = 13046	USNLSY USA *N* = 3657
Child Weight Status at Follow-Up^a^ (*n*, %)							
Not obese	2750 (69.0%)	935 (70.1%)	4663 (63.1%)	782 (57.7%)	3072 (75.2%)	9434 (72.3%)	1588 (43.4%)
Overweight	410 (10.3%)	244 (18.3%)	817 (11.1%)	262 (19.3%)	661 (16.2%)	2740 (21.0%)	556 (15.2%)
Obese	77 (1.9%)	85 (6.4%)	206 (2.8%)	99 (7.3%)	265 (6.5%)	872 (6.7%)	401 (11.0%)
Missing	747 (18.8%)	70 (5.2%)	1707 (23.1%)	213 (15.7%)	87 (2.1%)	0	1112 (30.4%)
Maternal Education at Baseline^b^ (*n*, %)							
High	1590 (39.9%)	463 (34.7%)	3191 (43.2%)	567 (41.8%)	1481 (36.3%)	4083 (31.3%)	1073 (29.3%)
Middle	2162 (54.3%)	536 (40.2%)	2035 (27.5%)	568 (41.9%)	2202 (53.9%)	5412 (41.5%)	1922 (52.6%)
Low	187 (4.7%)	336 (25.1%)	1488 (20.1%)	187 (13.8%)	400 (9.8%)	3068 (23.5%)	657 (18.0%)
Missing	45 (1.1%)	0	679 (9.2%)	34 (2.5%)	2 (0.1%)	483 (3.7%)	5 (0.01%)
Household Income at Baseline^c^ (*n*, %)							
High	912 (22.9%)	286 (21.4%)	1287 (17.4%)	365 (26.9%)	883 (21.6%)	2251 (17.3%)	570 (15.6%)
Middle	2471 (62.0%)	782 (58.6%)	2997 (40.5%)	874 (64.5%)	2524 (61.8%)	7523 (57.7%)	1581 (43.2%)
Low	597 (15.0%)	210 (15.7%)	1216 (16.4%)	117 (8.6%)	678 (16.6%)	2775 (21.3%)	825 (22.6%)
Missing	4 (0.1%)	56 (4.2%)	1893 (25.6%)	0	0	497 (3.8%)	681 (18.6%)
Child sex (*n*, %)							
Male	2101 (52.7%)	635 (47.6%)	3707 (50.1%)	687 (50.7%)	2096 (51.3%)	6592 (50.5%)	1881 (51.4%)
Female	1883 (47.3%)	699 (52.4%)	3685 (49.9%)	669 (49.3%)	1989 (48.7%)	6454 (49.5%)	1776 (48.6%)
Missing	0	0	1 (0.0%)	0	0	0	0
Mother ethnicity (*n*, %)							
Ethnic majority/Born in country	3739 (93.9%)	1224 (91.8%)	3967 (53.7%)	1232 (90.9%)	2650 (64.9%)	10 647 (81.6%)	2050 (56.1%)
Ethnic Minority/Born outside country	207 (5.2%)	109 (8.2%)	3168 (42.9%)	123 (9.1%)	1426 (34.9%)	1910 (14.7%)	1607 (43.9%)
Missing	38 (1.0%)	1 (0.1%)	258 (3.5%)	1 (0.1%)	9 (0.2%)	489 (3.7%)	0
Maternal Age at Child Birth^d^ (M, SD)	29.6 yr (4.64)	29.0 yr (5.1)	30.59 yr (5.10)	N/A	31.2 yr (5.2)	28.99 yr (5.99)	29.68 yr (3.12)

^a^Follow-up age varied by cohort: age 8–9 yrs in ABIS, LSAC; age 8–10 yrs in USNLSY; age 9–10 yrs in GenR; age 10–11 yrs in QLSCD, NLSCY, MCS.

^b^Maternal education harmonized across cohorts into 3 categories based on International Standard Classification of Education: high (ISCED V-VII), middle (ISCED III-IV), low (ISCED I-II).

^c^Household income grouped into high (5th quintile, richest), middle (2nd to 4th quintile), low (1st quintile, poorest).

^d^Maternal age at child birth was not available as a continuous variable for NLSCY; missing data reported for ABIS (*n* = 37, 0.9%), GenR (*n* = 1, 0.0%), LSAC (*n* = 3, 0.0%), USNLSY (*n* = 3, 0.0%).

(Unweighted estimates).

#### Income GINI index

Data on income inequality (GINI coefficient) after redistribution of countries at the baseline year of each cohort (or closest available) were derived from the World Bank. For Quebec, the GINI coefficient came from the local government’s estimates, see Table S2 (Supplementary data).

#### Statistical analysis

RRs were estimated using a generalized linear model with a log link and robust variance estimation with confounders entered as covariates in multivariate regressions [20]. Furthermore, RRs were weighted as follows: for two cohorts (ABIS, GenR), inverse probability weights were constructed to adjust for differential loss to follow-up using information on maternal education and income at baseline; five cohorts (QLSCD, NLSCY, LSAC, MCS, USNLSY) applied complex weights using additional variables (see Table S1, Supplementary data). Income and education were analysed separately in the multivariate analysis. A multivariate analysis with the SES measured simultaneously adjusted is presented in Table S6. Pooling of RRs and estimation of the Q and I^2^ statistics to evaluate heterogeneity were carried out using the R metafor package [21].

To evaluate the association between SES and childhood overweight and obesity on the absolute scale, we estimated the Slope Index of Inequality (SII) for each cohort [22]. The SII indicates the difference in prevalence of an outcome in the most advantaged group compared to the least advantaged. We used maternal education/household income group sizes and weighted prevalence estimates to calculate the SII for each inequality and outcome combination. While the estimated RRs allow us to compare the relative risk of overweight/obesity among groups, the SIIs indicate the absolute percentage of the population affected.

## Results

Prevalence among participants with data on height and weight ranged from 15.0% above the cut-off for overweight and 2.4% obese in the ABIS (Sweden) cohort to 37.6% overweight and 15.8% obese in the USNLSY (USA) cohort. Weighted prevalence of overweight and obesity by income and maternal education are shown in Table S3 (Supplementary data). The proportion of mothers from ethnic minority groups or born outside the cohort country was highest in the USNLSY (USA) cohort and lowest in ABIS (Sweden).

### Overweight

Unadjusted RRs of overweight by maternal education and household income groups, and confounding variables (child’s sex, mother ethnicity, maternal age) are shown in Table S4 (Supplementary material).

**Fig. 1:**
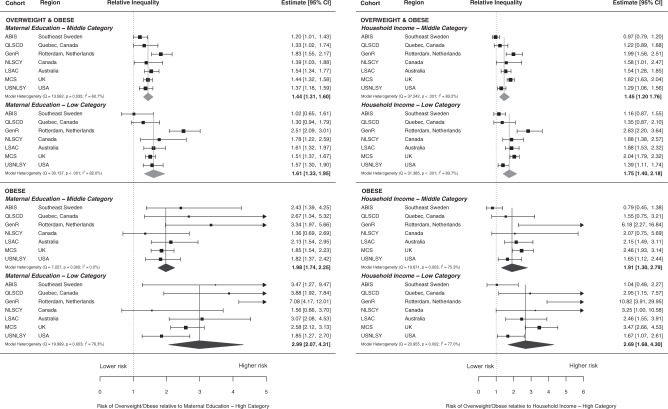
Forest plots of Overweight and Obesity by household income and maternal education. Upper left panel shows relative risks of Overweight for each individual cohort and pooled estimates in middle and low educational groups compared to high education. Lower left panel: relative risks of Obesity by education. Upper right panel: relative risks of Overweight by income. Lower right panel: relative risks of Obesity by income.

After adjustment for confounding variables, the RR of overweight comparing the lowest to the highest maternal education groups ranged from an RR of 1.02 (95% CI: 0.64, 1.60) in ABIS (Sweden) to 2.51 (95% CI: 2.09, 3.01) in GenR (Netherlands), see Table 2. There was significant heterogeneity in the observed maternal education-overweight associations across the different cohorts (heterogeneity *p* = 0.03 for middle education; *p* = <0.001 for low education), see Fig. 1. All cohorts showed increased RRs of overweight for lower maternal education level, although the confidence intervals around the estimate crossed unity in ABIS (Sweden) and QLSCD (Quebec, Canada). In two cohorts, ABIS (Sweden) and QLCSD (Quebec, Canada), the middle educational groups had the higher risk.

**Table 2:** Risk ratios for overweight/obese and obese at follow-up by household income and maternal education at baseline using adjusted multivariate regression.

	ABIS Sweden	QLSCD Quebec	GenR Netherlands	NLSCY Canada	LSAC-B Australia	MCS UK	USNLSY USA
Risk Ratio, (95% Confidence Interval)							
Overweight/Obese^a^							
Maternal Education^b^							
High (Reference)	1.00	1.00	1.00	1.00	1.00	1.00	1.00
Middle	1.20 (1.01, 1.43)	1.33 (1.02, 1.75)	1.83 (1.54, 2.16)	1.39 (1.03, 1.89)	1.54 (1.34, 1.78)	1.44 (1.31, 1.57)	1.37 (1.18, 1.59)
Low	1.02 (0.64, 1.60)	1.30 (0.94, 1.79)	2.51 (2.09, 3.01)	1.78 (1.22, 2.59)	1.61 (1.32, 1.97)	1.51 (1.38, 1.68)	1.57 (1.30, 1.90)
Household Income^c^							
High (Reference)	1.00	1.00	1.00	1.00	1.00	1.00	1.00
Middle	0.97 (0.79, 1.20)	1.22 (0.89, 1.68)	1.99 (1.58, 2.52)	1.45 (1.01, 2.46)	1.54 (1.28, 1.85)	1.82 (1.63, 2.04)	1.29 (1.06, 1.56)
Low	1.16 (0.87, 1.55)	1.35 (0.87, 2.10)	2.83 (2.20, 3.64)	1.88 (1.38, 2.57)	1.88 (1.53, 2.32)	2.04 (1.79, 2.32)	1.39 (1.11, 1.74)
Obese^a^							
Maternal Education^b^							
High (Reference)	1.00	1.00	1.00	1.00	1.00	1.00	1.00
Middle	2.43 (1.39, 4.25)	2.67 (1.34, 5.33)	3.34 (1.97, 5.66)	1.36 (0.69, 2.69)	2.13 (1.54, 2.95)	1.85 (1.54, 2.23)	1.82 (1.37, 2.42)
Low	3.47 (1.27, 9.45)	3.88 (1.92, 7.84)	7.08 (4.17, 12.00)	1.56 (0.66, 3.72)	3.07 (2.08, 4.53)	2.58 (2.12, 3.13)	1.85 (1.27, 2.71)
Household Income^c^							
High (Reference)	1.00	1.00	1.00	1.00	1.00	1.00	1.00
Middle	0.79 (0.45, 1.37)	1.55 (0.75, 3.22)	6.18 (2.27, 16.86)	2.07 (0.75, 5.67)	2.15 (1.49, 3.11)	2.46 (1.93, 3.14)	1.65 (1.12, 2.45)
Low	1.04 (0.48, 2.28)	2.95 (1.15, 7.57)	10.82 (3.91, 29.95)	3.25 (1.00, 10.60)	2.46 (1.55, 3.91)	3.47 (2.66, 4.53)	1.67 (1.07, 2.62)

^a^Adjusted for child sex, mother ethnicity, and maternal age at child birth. Maternal education and household income exposures at baseline. Overweight/Obese and Obese outcomes at follow-up; age varied by cohort: age 8–9 yrs in ABIS, LSAC; age 8–10 yrs in USNLSY; age 9–10 yrs in GenR; age 10–11 yrs in QLSCD, NLSCY, MCS.

^b^Maternal education harmonized across cohorts into three categories based on International Standard Classification of Education: high (ISCED V-VII), middle (ISCED III-IV), low (ISCED I-II).

^c^Household income grouped into high (5th quintile, richest), middle (2nd to 4th quintile), low (1st quintile, poorest). Values for 95% confidence intervals vary slightly versus those in the pooled forest plots due to rounding.

(Weighted estimates).

The RRs of the lowest income group compared to the highest for overweight ranged from 1.16 (95% CI: 0.87, 1.55) in ABIS (Sweden) to 2.83 (95% CI: 2.20, 3.64) in GenR (Netherlands). Heterogeneity between cohorts was significant for middle income (*p* = <0.001) and low income (*p* = <0.001). A trend in income gradient was observed in all cohorts except ABIS (Sweden); note that the confidence intervals crossed unity in ABIS (Sweden) and QLCSD (Quebec, Canada).

The pooled estimates of middle and low maternal education were 1.44 (95% CI: 1.31, 1.60) and 1.61 (95% CI: 1.33, 1.95), respectively, and for middle and low income they were 1.45 (95% CI: 1.20, 1.76) and 1.75 (95% CI: 1.40, 2.18). Forest Plots are shown in Fig. 1.

### Obesity

Unadjusted RRs of obesity by maternal education and household income groups and confounding variables (child’s sex, mother ethnicity, maternal age) are shown in Table S5 (Supplementary data).

After adjustment for potential confounders, the RR of obesity comparing the lowest to the highest maternal educational groups ranged from an RR of 1.56 (95% CI: 0.66, 3.72) in NLSCY (Canada) and 1.85 (95% CI: 1.27, 2.71) in USNLSY (USA) to 7.08 (95% CI: 4.17, 12.00) in GenR (Netherlands) see Table 2. There was no significant heterogeneity between cohorts for the relationship with middle education (*p* = 0.300); but, heterogeneity was observed with low education (*p* = 0.003). A social gradient by maternal education was present in all cohorts; note that the confidence intervals crossed unity in NLSCY (Canada).

For income and obesity, the RRs of the lowest income group compared to the highest income group ranged from RR 1.04 (95% CI: 0.48, 2.28) in ABIS (Sweden) to 10.82 (95% CI: 3.91, 29.95) in GenR (Netherlands). Heterogeneity between cohorts was significant for both middle income (*p* = 0.003) and low income (*p* = 0.002). A social gradient by income was present in all cohorts except ABIS (Sweden); note that the confidence intervals crossed unity in ABIS (Sweden).

The pooled estimates of middle and low maternal education were 1.98 (95% CI: 1.75, 2.25) and 2.99 (95% CI: 2.07, 4.31), respectively; and, for middle and low income, they were 1.91 (95% CI: 1.30, 2.79) and 2.69 (95% CI: 1.68, 4.30). Forest plots are shown in Fig. 1.

**Fig. 2:**
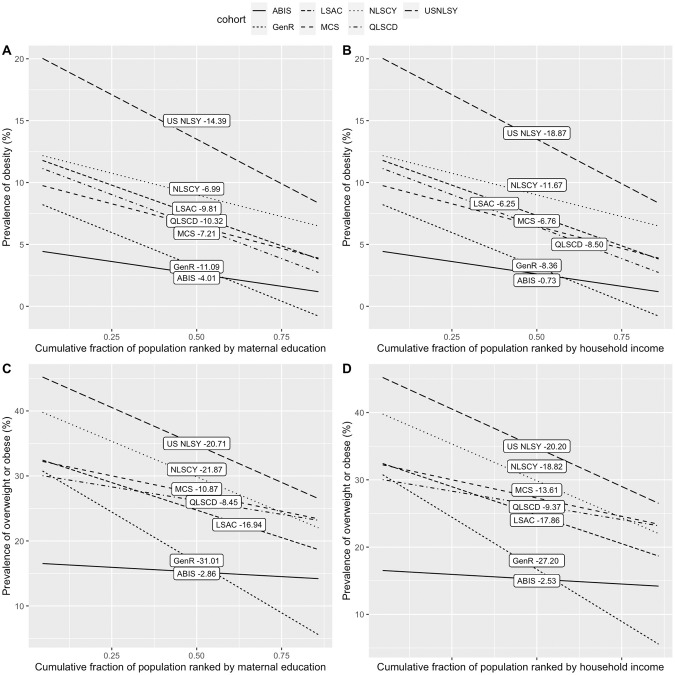
Slope index of inequalities (SIIs) plots of Overweight and Obesity by household income and maternal education. **A** Education and Obesity, **B** Income and Obesity, **C** Education and Overweight, **D** Income and Overweight.

The estimated SII by maternal education and income are illustrated for each cohort in Fig. 2. Absolute inequality in overweight/obesity across cohorts supports the lower risk for these adverse outcomes during late childhood in high income households or households with more highly educated mothers at birth or during early childhood. Absolute inequality in overweight by maternal education was most marked in NLSCY (Canada) at −21.87 and Gen-R (Netherlands) at −31.01 and least marked for ABIS (Sweden) at −2.86. For obesity by maternal education, USNLSY (USA) had the most marked inequality −14.39, while ABIS (Sweden) had the least marked inequality at −4.01. Absolute inequality by income for overweight was most marked in GenR (Netherlands) at −27.20 and USNLSY (USA) at −20.20 and least marked in ABIS (Sweden) at −2.53. For obesity by income, USNLSY (USA) had the most marked inequality at −18.87 and ABIS (Sweden) the least marked at −0.73. Previous research has found that absolute health inequality is related to income inequality (measured by the GINI coefficient) for some health outcomes [23]. However, a study on BMI in adolescents using family-affluence as measure of SES did not find an association between SII and income inequality among the 34 countries [12]. We illustrate how SII in overweight/obesity relates to income inequality in our seven cohorts for our two measures of SES (maternal education and income) in supplementary material, Fig. S1.

## Discussion

The findings in this study focused on the risk for child overweight and obesity at 8–11 years of age in different high-income country settings indicated that there is a SES gradient by maternal education in all participating cohorts. The evidence for a gradient by income was also strong, but heterogeneous. The weighted prevalence of childhood obesity ranging from 2.6% to 15.8% corresponds to previous cross-sectional studies [24, 25]. Our pooled RRs were also comparable with findings of a European study on maternal education and risk of overweight/obesity in children aged 4–7 years [10].

The absolute inequality measured by the SII supports the SES gradient in overweight/obesity across cohorts. The combination of relative inequality and absolute inequality provides a more complete account of the SES overweight/obesity relationship [26]. Absolute inequality also highlights the differences in prevalence between countries. In the Swedish ABIS cohort, low maternal education yielded a high risk of obesity in relative terms (RR:3.47; CI: 1.27, 9.45), but the absolute inequality (SII: −4.01; CI −4.08, −3.93) was lower than in all other cohorts. Houweling TA et al observed that relative inequality tends to be more pronounced when the health outcome is less prevalent, while the relationship between absolute inequality and prevalence takes the shape of an inverse U, being lowest at low and high prevalence [27]. Our study shows that for overweight/obesity in children these patterns only holds to some extent. The ABIS (Sweden) cohort with the lowest prevalence did indeed have low absolute inequality. However, the results for relative inequality in ABIS were mixed with a high RR for the maternal education and obesity association but low RR for the association between income and obesity and low RRs for the associations between both SES measures and overweight. The GenR (Netherlands) cohort, which had the second lowest prevalence of obesity, also deviated from the findings of Houweling TA et al. by showing that high absolute inequality can be found also in a low prevalence setting. The other cohorts from Quebec, Australia, Canada and UK with intermediate prevalence showed rather similar patterns of intermediate relative and absolute inequality. The USNLSY (USA) cohort with the highest prevalence of overweight/obesity also had low to intermediate levels of relative inequality but high absolute inequality. This observation was consistent with the proposal of Houweling TA et al. General patterns of overweight and obesity inequalities in relation to prevalence are important when comparing inequality levels across countries. However, our study shows that these general patterns do not fully explain the observed differences, an explanatory theory should account for other factors such as social and economic policy differences across countries.

Low RRs for the association between overweight and both SES measures and low RR for the obesity-low income association, but high RR for the obesity-low maternal education association was observed for the ABIS cohort (Sweden). This observation stood in contrast to other cohorts, where both low maternal education and low income were association with higher risk of overweight than in Sweden and the obesity-income association were higher. Studies focused on the risk of developing overweight/obesity in late childhood in relation to early childhood SES using both maternal education and household income are rare. However, in previous Scandinavian studies of children aged 7 years from Denmark, and aged 15–16 years from Norway, maternal education was found to be more closely associated with obesity than income [28, 29]. Studies from the US (all using highest education of either parent) found a stronger association with income than with education [30], or strong association with both measures of SES [31].

Sweden has social and family policies that we hypothesize may explain the differences in RR by income between cohorts found in our study. Four policies differed between Sweden versus the other national cohorts (Netherlands, Canada, Australia, UK, USA): (i) universal preschools, (ii) generous parental leave regulations, (iii) a ban on advertising to children, and (iv) universal free school meals.

A universal preschool policy commonly reduces the impact of low household income by strengthening the economic situation of low SES families as preschool costs are a significant part of household income in countries that do not have state subsidy [32]. An ecological study across 35 OECD countries found that social spending on preschools was associated with reduced child obesity prevalence [13]. A universal preschool policy that increases attendance of children from low-income families could, therefore, potentially reduce SES disparities in overweight/obesity.

The reduced associations observed between income and child overweight/obesity in Sweden compared to other cohorts, could also be related to parental leave regulations. Income is plausibly less strongly correlated with education in Sweden because parents with higher education, including fathers, tend to take longer parental leave. Relatedly, parental leave reduces income because the subsidized benefit only compensates a portion of the household salary and has an upper limit of compensation [33].

A ban on advertisements aimed at children was adopted in Sweden during the cohort timeframes. The effectiveness of these regulations has decreased, starting in 1997 with EU regulations that made it possible for TV channels broadcasting from the UK to avoid the Swedish regulations and followed later by internet-based media that bypasses Swedish law entirely [34]. Notably, similar advertising restrictions existed for Quebec although the RR pattern is dissimilar from Sweden. Still, these regulations may partly explain the observed difference between the participating cohorts in our study as a reduction in advertisements aimed at children has been shown to be associated with a reduction in overweight/obesity, especially in children from disadvantaged areas [35].

A fourth child health policy, unique to Sweden during our study time-period, that may explain our observed differences in SES-overweight/obesity is free school meals. Universal free school meals were adopted as early as 1946 in Sweden. Recently, universal free school lunches have been shown to reduce obesity, with a stronger effect in low SES children, when introduced in primary schools in the UK [15]. An evaluation of the long-term effects of free school lunches to all children in Sweden has shown positive effects on children´s economic, educational and health outcomes throughout life [36].

Quebec today shares all the policies adopted by Sweden except universal school meals, however, at the time of the start of our Quebec cohort (1997) low cost preschools had been implemented only the same year and parental leave including income insurance was not implemented until in 2006, see supplementary policy table. Only the ban on advertisement, introduced in 1980 in Quebec, was in full effect. Studies on the effect of low cost preschools introduced in 1997 in Quebec have found that the effect of that policy came gradually [37]. Future studies are necessary to determine if the low income-obesity association seen in Sweden will appear also in Quebec once these policies have their full effect; if so, this would strengthen the argument for a probable causal relationship.

The low obesity prevalence with high inequality both in relative and absolute terms for GenR (Netherlands) might seem unexpected given the Netherlands’ fairly low-income inequality (GINI) level. However, previous cross-sectional WHO studies in the Netherlands reported similar findings with low prevalence and high levels of inequality, especially among boys (using family affluence as the measure of SES) [38]. In our study, RRs and absolute risk were marked especially for income; this was explained by a very low prevalence of childhood obesity in the high-income group (0.5%). The Netherlands have been very successful in enabling active transport (bicycling) in both children and adults, which may explain the low total prevalence. However, additional policies are required to reduce overweight/obesity among low SES children.

There is evidence of a relationship between the GINI coefficient higher risk of overweight/obesity in children, and lower physical activity and higher BMI-z scores in adolescent [11, 12]. Our results are largely aligned with these findings; the countries with the lowest income GINI-coefficients (Sweden and the Netherlands) were also the countries with the lowest overweight/obesity prevalence. Of note, the Quebec cohort did not follow this pattern, despite having an income GINI slightly lower than the Netherlands, with overweight/obesity rates more comparable to Canada, Australia, and the UK. Moreover, as illustrated in Supplementary Fig. S1, our data indicates a plausible relationship between absolute inequality (SII measured by income) in obesity and the GINI coefficient. However, with only seven cohort data points, our study power is insufficient to draw firm conclusions regarding this relationship. Further studies comparing additional countries and accounting for other important country-level factors (e.g., medical/social spending, employment rate) are necessary to investigate this relationship.

### Strengths and limitations

A major strength of this study was the prospective design of the included child cohorts from six high-income countries. This design allowed us to investigate the social and economic circumstances of the families in early childhood and their associations with later childhood risk of overweight/obesity. Harmonization of SES measures was performed for each the cohort. Non-participation and differential attrition were accounted for by weighting and/or imputation in all cohorts except the US cohort. This study reported RRs rather than ORs, avoiding the issues of non-collapsibility of the latter measure and providing easily interpretable metrics of risk. This is especially true for overweight, which is a prevalent outcome for which the OR is no longer a good estimate of the RR [9].

Several limitations should be considered when interpreting the findings. First, not all cohorts had data on after-tax income; a standardized adjustment was used to account for this. Second, parental-report of child height and weight (in ABIS, NLSCY, and USNLSY cohorts) could have introduced reporter bias; however, previous studies are inconsistent on the extent and direction of SES related parental reporting bias of overweight/obesity [39, 40]. We cannot rule out that the use of parental-report might have biased the results slightly in either direction in the studies that used this method for obtaining height and weight, but the implications for the interpretation of the result should be limited [40]. Third, the Dutch Generation R study is conducted within the Rotterdam area, with higher income inequality than the Netherlands as a whole; this may have affected the external validity of the results. Fourth, disadvantaged families in the US cohort were likely to be under-represented because it did not include children of young mothers [41]. Finally, the baseline year differed across cohorts with most cohorts starting around 2000, but with a range between 1988–96 (USNLSY, USA) to 2004 (GenR, Netherlands; LSAC, Australia). Given the temporal trend in childhood overweight/obesity with increasing prevalence over time, this difference does affect the comparison of prevalence between cohorts.

## Conclusions

There is clear evidence for a social gradient by maternal education in early childhood in the relative risk of childhood obesity among the high income countries included in this study. The potential associations of SES measured by household income were more heterogeneous and differed particularly between Sweden and the other cohorts. The differences in the income - overweight/obesity associations between cohorts might be related to differences in social and family-policies, including universal preschool, parental leave policies, regulations on advertisement to children, and universal free school meals.

### Data sharing

Data underlying the results presented in this EPOCH study are available from the primary data sources. Data from the UK Millennium Cohort Study is available in a public open-access repository (https://cls.ucl.ac.uk/cls-studies/millennium-cohortstudy/). Data from the Longitudinal Study of Australian Children (LSAC) is available in a public, open-access repository (https://growingupinaustralia.gov.au/data-anddocumentation). Data from the US NLSY-79 is available in a public open-access repository (https://www.nlsinfo.org/content/cohorts/nlsy79-children). Data from the Rotterdam, Netherlands Generation R are available to request from (https://generationr.nl/researchers/); authors do not have permission to share their data. Data from the Sweden Alla Barn I Sydöstra Sverige (ABIS) are available to request from (http://www.abis-studien.se); authors do not have permission to share their data. Data from the Quebec Longitudinal Study of Child Development (QLSCD) is available to request from (https://www.maelstrom-research.org/mica/individualstudy/qlscd#); authors do not have permission to share their data.

## Supplementary information

EPOCH Obesity Supplementary information

EPOCH Obesity Supplementary tables

EPOCH Obesity Supplementary Figure 1 SII and GINI

## References

[1] Abarca-Gómez L, Abdeen ZA, Abdul Hamid Z, Abu-Rmeileh NM, Acosta-Cazares B, Acuin C (2017). Worldwide trends in body-mass index, underweight, overweight, and obesity from 1975 to 2016: a pooled analysis of 2416 population-based measurement studies in 128.9 million children, adolescents, and adults. Lancet.

[2] Buoncristiano M, Spinelli A, Williams J, Nardone P, Rito AI, García-Solano M (2021). Childhood overweight and obesity in Europe: Changes from 2007 to 2017. Obes Rev.

[3] Stierman B, Ogden CL, Yanovski JA, Martin CB, Sarafrazi N, Hales CM (2021). Changes in adiposity among children and adolescents in the United States, 1999-2006 to 2011-2018. Am J Clin Nutr.

[4] Chung A, Backholer K, Wong E, Palermo C, Keating C, Peeters A (2016). Trends in child and adolescent obesity prevalence in economically advanced countries according to socioeconomic position: a systematic review. Obes Rev.

[5] Braddon FE, Rodgers B, Wadsworth ME, Davies JM (1986). Onset of obesity in a 36 year birth cohort study. Br Med J (Clinical research ed.).

[6] Sobal J, Stunkard AJ (1989). Socioeconomic status and obesity: a review of the literature. Psychol Bull.

[7] Shrewsbury V, Wardle J (2008). Socioeconomic status and adiposity in childhood: a systematic review of cross-sectional studies 1990-2005. Obesity (Silver Spring, Md.).

[8] Wu S, Ding Y, Wu F, Li R, Hu Y, Hou J (2015). Socio-economic position as an intervention against overweight and obesity in children: a systematic review and meta-analysis. Sci Rep.

[9] Cummings P (2009). The relative merits of risk ratios and odds ratios. Arch Pediatr Adolesc Med.

[10] Ruiz M, Goldblatt P, Morrison J, Porta D, Forastiere F, Hryhorczuk D (2016). Impact of low maternal education on early childhood overweight and obesity in Europe. Paediatr Perinat Epidemiol.

[11] Pickett KE, Wilkinson RG (2007). Child wellbeing and income inequality in rich societies: ecological cross sectional study. BMJ.

[12] Elgar FJ, Pfortner TK, Moor I, De Clercq B, Stevens GW, Currie C (2015). Socioeconomic inequalities in adolescent health 2002-2010: a time-series analysis of 34 countries participating in the Health Behaviour in School-aged Children study. Lancet.

[13] Miyawaki A, Evans CEL, Lucas PJ, Kobayashi Y (2021). Relationships between social spending and childhood obesity in OECD countries: an ecological study. BMJ Open.

[14] Wang Y, Cai L, Wu Y, Wilson RF, Weston C, Fawole O (2015). What childhood obesity prevention programmes work? A systematic review and meta-analysis. Obes Rev.

[15] Holford AR,B The impact of the Universal Infant Free School Meal policy Institute for Social and Economic Research, University of Essex.

[16] Sadeghirad B, Duhaney T, Motaghipisheh S, Campbell NR, Johnston BC (2016). Influence of unhealthy food and beverage marketing on children’s dietary intake and preference: a systematic review and meta-analysis of randomized trials. Obes Rev.

[17] Pan X, Zhao L, Luo J, Li Y, Zhang L, Wu T (2021). Access to bike lanes and childhood obesity: A systematic review and meta-analysis. Obes Rev.

[18] Ventura SJ, Hamilton BE, Matthews TJ (2014). National and state patterns of teen births in the United States, 1940–2013. Natl Vital Stat Rep.

[19] Cole TJ, Lobstein T (2012). Extended international (IOTF) body mass index cut-offs for thinness, overweight and obesity. Pediatric Obesity.

[20] Zou GY, Donner A (2013). Extension of the modified Poisson regression model to prospective studies with correlated binary data. Stat Methods Med Res.

[21] Viechtbauer W (2010). Conducting meta-analyses in R with the metafor package. J Stat Softw.

[22] Health Equity Assessment Toolkit (HEAT) In. 2.0 ed. Geneva: World Health Organization, 2017. pp Software for exploring and comparing health inequalities in countries. Built-in database edition. 10.1186/s12874-016-0229-9 PMC5069829 27760520

[23] Wilkinson RG, Pickett KE (2008). Income inequality and socioeconomic gradients in mortality. Am J Public Health.

[24] Haug E, Rasmussen M, Samdal O, Iannotti R, Kelly C, Borraccino A (2009). Overweight in school-aged children and its relationship with demographic and lifestyle factors: results from the WHO-Collaborative Health Behaviour in School-aged Children (HBSC) study. Int J Public Health.

[25] Ogden CL, Carroll MD, Lawman HG, Fryar CD, Kruszon-Moran D, Kit BK (2016). Trends in obesity prevalence among children and adolescents in the United States, 1988–1994 through 2013–2014. Jama.

[26] King NB, Harper S, Young ME (2012). Use of relative and absolute effect measures in reporting health inequalities: structured review. BMJ.

[27] Houweling TA, Kunst AE, Huisman M, Mackenbach JP (2007). Using relative and absolute measures for monitoring health inequalities: experiences from cross-national analyses on maternal and child health. Int J Equity Health.

[28] Morgen CS, Andersen PK, Mortensen LH, Howe LD, Rasmussen M, Due P (2017). Socioeconomic disparities in birth weight and body mass index during infancy through age 7 years: a study within the Danish National Birth Cohort. BMJ Open.

[29] Lien N, Kumar BN, Holmboe-Ottesen G, Klepp KI, Wandel M (2007). Assessing social differences in overweight among 15- to 16-year-old ethnic Norwegians from Oslo by register data and adolescent self-reported measures of socio-economic status. Int. J Obesity.

[30] Hansstein FV (2016). The impact of breastfeeding on early childhood obesity: evidence from the national survey of children’s health. Am J Health Promot.

[31] Kimm SY, Obarzanek E, Barton BA, Aston CE, Similo SL, Morrison JA (1996). Race, socioeconomic status, and obesity in 9- to 10-year-old girls: the NHLBI Growth and Health Study. Ann Epidemiol.

[32] OECD (2020), “Is Childcare Affordable?” Policy Brief on Employment, Labour and Social Affairs, OECD, Paris, oe.cd/childcare-brief-2020.

[33] Eriksson H (2019). Taking turns or halving it all: care trajectories of dual-caring couples. Eur J Popul.

[34] Cathaoir KÓ (2017). Food marketing to children in sweden and denmark: a missed opportunity for nordic leadership. Eur J Risk Regul.

[35] Brown V, Ananthapavan J, Veerman L, Sacks G, Lal A, Peeters A (2018). The potential cost-effectiveness and equity impacts of restricting television advertising of unhealthy food and beverages to Australian children. Nutrients.

[36] Lundborg P, Rooth D-O, Alex-Petersen J. Long-term effects of childhood nutrition: evidence from a school lunch reform. Rev Economic Studies. 2021.

[37] Haeck C, Lefebvre P, Merrigan P (2015). Canadian evidence on ten years of universal preschool policies: The good and the bad. Labour Economics.

[38] Schokker DF, Visscher TLS, Nooyens ACJ, Van Baak MA, Seidell JC (2007). Prevalence of overweight and obesity in the Netherlands. Obes Rev.

[39] Weden MM, Brownell PB, Rendall MS, Lau C, Fernandes M, Nazarov Z (2013). Parent-reported height and weight as sources of bias in survey estimates of childhood obesity. Am J Epidemiol.

[40] Brettschneider AK, Ellert U, Schaffrath Rosario A (2012). Comparison of BMI derived from parent-reported height and weight with measured values: results from the German KiGGS study. Int J Environ Res Public Health.

[41] Hobcraft J, Kiernan K (2001). Childhood poverty, early motherhood and adult social exclusion. Br J Sociol.

