# Supplementary material for: Household income and maternal education in early childhood and risk of overweight and obesity in late childhood: Findings from seven birth cohort studies in six high-income countries
Source: Int J Obes (Lond). 2022 Jul 11;46(9):1703–11. doi: 10.1038/s41366-022-01171-7 (PMC9395266; doi:10.1038/s41366-022-01171-7)
Supplement: Supplementary file 1 — EPOCH Obesity Supplementary information [file 41366_2022_1171_MOESM1_ESM.docx]

**Supplementary information**

**Household income and maternal education in early childhood and risk of overweight and obesity in late childhood: findings from seven birth cohort studies in six high-income countries**

***Acknowledgement***

Sincere thanks to the dedicated EPOCH administrative staff, especially Sabrina Giovanniello and Julie Foisy (Research Coordinators; Concordia University, Canada), without whom this research would not be possible. We are grateful to all families who participated in All Babies in Southeast Sweden (ABIS), Generation R Study (GenR), Longitudinal Study of Australian Children (LSAC), UK Millennium Cohort Study (MCS), National Longitudinal Study of Children and Youth (NLSCY), Québec Longitudinal Study of Child Development (QLSCD), and US National Longitudinal Survey of Youth (US-NLSY79) birth cohorts.

Generation R Study (GenR) is conducted by Erasmus Medical Center in close collaboration with the School of Law and Faculty of Social Sciences of the Erasmus University Rotterdam, the Municipal Health Service Rotterdam area, Rotterdam, the Rotterdam Homecare Foundation, Rotterdam and the Stichting Trombosedienst & Artsenlaboratorium Rijnmond (STAR-MDC), Rotterdam; we gratefully acknowledge the contribution of children and parents, general practitioners, hospitals, midwives and pharmacies in Rotterdam.

UK Millennium Cohort Study (MCS) was led by Centre for Longitudinal Studies at the Institute of Education of the University of London; we thank the Economic and Social Data Service and the United Kingdom Data Archive for permission to access study data.

National Longitudinal Study of Children and Youth (NLSCY) acknowledges these analyses were based on Statistics

Canada master files for NLSCY Cycles 1–7, which contain anonymized data collected from 1994 to 2007. The responsibility for the use and interpretation of these data is solely that of the authors. The opinions expressed in this article are those of the authors and do not represent the view of Statistics Canada.

US National Longitudinal Survey of Youth (US-NLSY79) is managed by the Center for Human Resource Research at The Ohio State University and interviews are conducted by the National Opinion Research Center at the University of Chicago.

***Funding***

EPOCH was partly supported by Canadian Institutes of Health Research (J. McGrath OCO-79897, MOP-89886, MSH- 95353; L. Séguin ROG-110537). ABIS and this research were supported in part by the County Council of Ostergotland, Swedish Research Council (K2005-72X-11242-11A and K2008-69X-20826-01-4), the Swedish Child Diabetes Foundation (Barndiabetesfonden), Juvenile Diabetes Research Foundation, Wallenberg Foundation (K 98-99D-12813-01A), Medical Research Council of Southeast Sweden (FORSS), the Swedish Council for Working Life and Social Research (FAS2004–1775), and Ostgota Brandstodsbolag. Johnny Ludvisson founded the ABIS Cohort. Longitudinal Study of Australian Children (LSAC) was initiated and funded by Australian Government Department of Social Services, with additional funding from partner organizations Australian Institute of Family Studies (AIFS) and Australian Bureau of Statistics (ABS). The study was conducted in partnership with the Department of Social Services (DSS), the Australian Institute of Family Studies (AIFS) and the Australian Bureau of Statistics (ABS). The findings and views reported in this paper are those of the authors and should not be attributed to the DSS, the AIFS or the ABS. This paper uses unit record data from Growing Up in Australia, the Longitudinal Study of Australian Children. Generation R Study (GenR) was made possible by financial support from Erasmus Medical Center, Rotterdam; Erasmus University Rotterdam; Netherlands Organisation for Health Research and Development (ZonMw; additional grant received by V. Jaddoe, ZonMw 907.00303, 916.10159); Netherlands Organisation or Scientific Research (NWO); Ministry of Health, Welfare and Sport; and, Ministry of Youth and Families. Generation R Study (GenR) is conducted by Erasmus Medical Center in close collaboration with the School of Law and Faculty of Social Sciences of the Erasmus University Rotterdam, the Municipal Health Service Rotterdam area, Rotterdam, the Rotterdam Homecare Foundation, Rotterdam and the Stichting Trombosedienst & Artsenlaboratorium Rijnmond (STAR-MDC), Rotterdam; we gratefully acknowledge the contribution of children and parents, general practitioners, hospitals, midwives and pharmacies in Rotterdam. Québec Longitudinal Study of Child Development (QLSCD) 1996-2014 cohort was principally funded and supported by l’Institut de la statistique du Québec through partnership with Fondation Lucie et André Chagnon, Ministe re de l’Éducation et de l’Enseignement supérieur, Ministe re de la Santé et des Services sociaux, Ministere de la Famille, GRIP Research Unit on Children’s Psychosocial Maladjustment, QUALITY Cohort Collaborative Group, le Centre hospitalier universitaire Sainte- Justine, Institut de recherche Robert-Sauvé en santé et en securité au travail, l’Institut de recherche en santé publique de l’Université de Montréal, Centre de recherche du Centre hospitalier de l’Université de Montréal (CRCHUM), Fonds de recherche du Québec Santé (FRQS), Fonds de recherche du Québec Sociéte et culture (FRQSC), Social Sciences and Humanities Research Council (SSHRC), and Canadian Institutes of Health Research (MOP-123079, HDF-70335). The paper used unit record data from the QLSCD (ELDEQ – Enquête longitudinale des enfants du Québec). Data for the QLSCD were collected by the Institut de la Statistique du Québec, Direction des enquêtes longitudinales et sociales. National Longitudinal Study of Children and Youth (NLSCY) was conducted by Statistics Canada and sponsored by Human Resources and Skills Development Canada (HRSDC); both agencies played a role in funding, development of survey content, research, and dissemination of findings. NLSCY and this research was supported by funds to the Canadian Research Data Centre Network (CRDCN) from the Social Sciences and Humanities Research Council (SSHRC), the Canadian Institute for Health Research (CIHR), the Canadian Foundation for Innovation (CFI), and Statistics Canada. Although the research and analysis are based on data from Statistics Canada, the opinions expressed do not represent the views of Statistics Canada. The UK Millennium Cohort Study (MCS) was supported by the Economic and Social Research Council, the Office of National Statistics, and various government departments. The study was led by the Centre for Longitudinal Studies at the Institute of Education of the University of London. We thank the Economic and Social Data Service and the United Kingdom Data Archive for permission to access the study data. The US National Longitudinal Survey of Youth (US-NLSY79) is sponsored and directed by U.S. Bureau of Labor Statistics and conducted by Center for Human Resource Research at The Ohio State University. Interviews are conducted by the National Opinion Research Center (NORC) at the University of Chicago. The Children of the NLSY79 survey is sponsored and directed by the U.S. Bureau of Labor Statistics and the National Institute for Child Health and Human Development.

***Ethical approval***

The authors assert that all procedures contributing to this work involving human participants comply with the ethical standards of the relevant institutional and/or national committees and with the Helsinki Declaration of 1964, and its later amendments or comparable ethical standards.

Concordia University Human Research Ethics Committee certified the ethical acceptability for the EPOCH project (secondary use of multiple sources of anonymous data; #2011028).

All Babies in Southeast Sweden (ABIS) initially approved by Regional Ethic Committee at Lunds University (Dnr 83-97) and Regional Ethic Committee at Linköping University (Dnr Li287-96); subsequent sub-studies approved by Regional Ethic Committee at Linköping University (Dnr 2003/513; Dnr 2013/253-32).

Generation R Study general design, research aims, and specific measurements were approved by Medical Ethical Committee of the Erasmus Medical Center, Rotterdam. Longitudinal Study of Australian Children (LSAC) was approved by Australian Institute of Family Studies (AIFS) Ethics Committee.

UK Millennium Cohort Study (MCS) received ethics approval from South West Multi-Centre Research Ethics Committee, London Multi- Centre Research Ethics Committee of the National Health Service Ethical Authority (NHS), & Northern and Yorkshire Multi-Centre Research Ethics Committee of the NHS (MCS1 MREC/01/6/19; MCS2 MREC/03/2/022; MCS3 05/MRE02/46; MCS4 07/MRE03/32) and Yorkshire and Humber REC (MCS5 Ref: 11/YH/0203).

The substudy of the QLSCD (Enquête longitudinale des enfants du Québec – ELDEQ) data in the context of the EPOCH project was approved by the Comité d’éthique à la recherche du Centre Hospitalier de l’Université de Montréal (#CE 13.105).

In addition to the U.S. Office of Management and Budget, the US NLSY79 survey was reviewed and approved by the institutional review boards at the institutions that manage and conducted the surveys under contract with the U.S. Bureau of Labor Statistics, including The Ohio State University, and the National Opinion Research Center (NORC) at the University of Chicago.

The National Longitudinal Survey of Children and Youth was developed jointly by Statistics Canada and Human Resources Development Canada. Statistics Canada carried out this national study on behalf of Human Resources Development Canada.

***Data sharing***

Data underlying the results presented in this EPOCH study are available from the primary data sources. Data from the UK Millennium Cohort Study is available in a public open-access repository (<https://cls.ucl.ac.uk/cls-studies/millennium-cohortstudy/>). Data from the Longitudinal Study of Australian Children (LSAC) is available in a public, open-access repository (<https://growingupinaustralia.gov.au/data-anddocumentation>). Data from the US NLSY-79 is available in a public open-access repository (https://www.nlsinfo.org/content/cohorts/nlsy79-children). Data from the Rotterdam, Netherlands Generation R are available to request from (https://generationr.nl/researchers/); authors do not have permission to share their data. Data from the Sweden Alia Barn I Sydöstra Sverige (ABIS) are available to request from (http://www.abis- studien.se); authors do not have permission to share their data. Data from the Quebec Longitudinal Study of Child Development (QLSCD) is available to request from (https://www.maelstrom-research.org/mica/individualstudy/qlscd#); authors do not have permission to share their data.
