# Supplementary material for: Household income and maternal education in early childhood and risk of overweight and obesity in late childhood: Findings from seven birth cohort studies in six high-income countries
Source: Int J Obes (Lond). 2022 Jul 11;46(9):1703–11. doi: 10.1038/s41366-022-01171-7 (PMC9395266; doi:10.1038/s41366-022-01171-7)
Supplement: Supplementary file 2 — EPOCH Obesity Supplementary tables [file 41366_2022_1171_MOESM2_ESM.docx]

Supplementary tables

**Supplementary Table S1: Cohort Profiles**

| **Cohort Country/Region**  **Start date** | **Sampling Methodology** | **Age & Sample Size**  **Attrition** | **Household Income** | **Child Height  & Weight** | **Mother Ethnicity Definition** | **Weighting & Imputation**  **for Attrition** |
| --- | --- | --- | --- | --- | --- | --- |
| ABIS  Southeast Sweden  1997-1999 | All children born October 1, 1997 to September 30, 1999, in a defined region in southeast of Sweden were invited | Baseline: Birth  N=17055  Follow-up: 8/9 yrs  N=3984    Attrition: 76.6% | Net of tax  (National register)  Child age: 1-3 yrs | Anthropometric measures by parental-report  Child age: 8/9 yrs | Mother born in country  (*Yes* OR *No*) | Weights applied for loss to follow-up  No imputation |
| QLSCD  Quebec, Canada  1997 | All singleton life births, born in 1997 from mothers living in Quebec, except in First Nation’s territories. Excluded very premature or post-term birth, and when sex or gestational age were unknown | Baseline: 6 mos  N=2120  Follow-up: 10/11 yrs  N=1334  Attrition: 37% | Gross income  (Income one year before maternity leave)  Child age: 6 mos | Anthropometric measures taken by nurse  Child age: 10/11 yrs | Mother born in country (*Yes* OR *No*) | Weights applied (transversal to adjust for participation in a given wave; longitudinal to account for differential attrition)  No imputation |
| GenR The Netherlands, Rotterdam 2002-2004 | All eligible pregnant women who visited a midwife or obstetrician in Rotterdam were contacted by Generation R Study staff | Baseline: 2 mos  N=9749  Follow-up: 9-10 yrs  N=7393  Attrition: 24.2% | Net of tax  Child age: 5 yrs | Anthropometric measures taken by research assistant  Child age: 9-10 yrs | Mother ethnic group  (*Majority/Dutch* OR *Minority*) | Weights applied for loss to follow-up  No imputation |
| NLSCY  Canada  1994 | National sample stratified by province | Baseline: 0–11 mos  N=2227  Follow-up: 10-11 yrs  N=1356  Attrition: 39.1% | Gross income  Child age: 0-11 mos | Anthropometric measures by parental report  Child age: 10-11 yrs | Mother born in country  (*Yes* OR *No*) | Weights applied to account for differential attrition and to weight back to the population  No imputation |
| LSAC-B  Australia  2004 | National sample using two-stage random sampling design: (1) random selection of 10% of postcodes (stratified by state and urban/rural locations), (2) random selection of in-age children within those postcodes from Medicare (universal healthcare) database. Very remote postcodes and those with <20 children (n=874 postcodes, 3.2% of population)  were excluded. | Baseline: 0-1 yrs N=5107  Follow-up: 8-9 yrs  N=4085  Attrition: 20.0% | Gross income  Child age: 0-1 yrs | Anthropometric measures taken by research assistant  Child age: 8-9 yrs | Mother born in country  (*Yes* OR *No*) | Weighted back to the reference population (target of sample design)  Weights adjusted for initial non-response and subsequent loss to follow-up |
| MCS  UK  2000 | All children born between September 1, 2000 and August 31, 2001 (for England & Wales), and between November 24, 2000 and January 11, 2002 (for Scotland and Northern Ireland), alive and living in the UK at age 9 mos and eligible to receive child benefit at that age. Eligibility based on government child benefit records (i.e., nearly universal coverage); asylum seekers not eligible. Subgroups intentionally oversampled (living in disadvantaged areas, ethnic minorities) | Baseline: 9 mos  N=18,552  Follow-up: 10-11 yrs N=13,354  Attrition: 28.4% | Net of tax  Child age: 9 mos | Anthropometric measures taken by research assistant  Child age: 10/11 yrs | Mother ethnic group  (*Majority* OR *Minority*) | Weights applied  Imputation for differential attrition |
| USNLSY  USA  1988-1996 | Original NLSY79 cohort was cross-sectional, population representative sample born between January 1958 and December 1964; subsamples intentionally oversampled Hispanic or Latino, Black, economically disadvantaged non-Black/non-Hispanic, and military personnel. NLSY79 Child and Young Adult cohort follows offspring born to female respondents of the original NLSY79 cohort. Analytical sample for present study was limited to children born between 1988 to 1996. | Baseline: Birth  N=3657  Follow-up: 8-10 yrs  N=2545  Attrition: 30.4% | Net of tax  Child age: 0-2 yrs | Anthropometric measures taken by research assistant or parental report  Child age: 8-10 yrs | Mother of ethnic group  (*Majority/White, non-Hispanic* OR *Minority*) | Weights applied to account for differential attrition and to weight back to the population  No imputation |

**Supplementary Table S2: Ethnic and Family Policies by Cohort Jurisdiction**

|  | **Sweden** | **Quebec**  **(Canada)** | **The Netherlands** | **Canada**  **(all Provinces)** | **Australia** | **United  Kingdom** | **USA** |
| --- | --- | --- | --- | --- | --- | --- | --- |
|  | ABIS | QLSCD | GenR | NLSCY | LSAC-B | MCS | USNLSY |
| Income Inequality (Gini-coefficient) | 27.2 | 29.5 | 29.8 | 31.3 | 33.1 | 37.0 | 40.0 |
| Year (closest to cohort baseline with data available^1^) | 2000 | 1998^2^ | 2004 | 1994 | 2004 | 2000 | 1994 |
| Paid Maternity Leave (weeks) 2000^1^ | 58.7 | 15^4^  (10 shared with father) | 16 | 27 | 0 | 18 | 0 |
| Paid Paternity Leave (weeks) 2000^3^ | 5.8 | 0^4^  (10 shared with mother) | 0 | 0 | 0 | 0 | 0 |
| Paid Leave for Mothers (weeks) 2017^3^ | 35 | 30.0 to 31.3 (Introduced in 2006)^5a^ | 16 | 27 | 8 | 12 | 0 |
| Paid Leave for Fathers (weeks) 2017^3^ | 10.9 | 21.0 to 22.2^5b^ | 0.4 | 0 | 0.8 | 0.4 | 0 |
| Relative Income Poverty for Children (0-17 yrs) (%)^6^ | 9.1% | 14.3%^7^ | 10.4% | 19.9% | 17.1% | 11.2% | 13.0% |
| Maternal Employment Rate (%)^6^ | 83.1% | 78.6%^8^ | 74.8% | 65.7% | 73.7% | 67.1% | 62.9% |
| Daycare / Preschool Cost (Percentage of household income, family with 2 children; 2004) (%)^9^ | 7% | 5%^10^ | 18% | 27% | 21% | 39% | 29% |
| Free School Meals during cohort time period | Yes  (All children, since 1973)^11^ | Eligible children only | No | No | No | Eligible children only^11^  (Grades R, 1, 2, since 2014) | Eligible children only^12^ |
| Ban on Food Advertisement to Children | Yes  (since 1991)^13^ | Yes  (since 1980)^14^ | No | Self-regulated codes^15^ | No | Yes  (since 2007)^14^ | No |
| Physical Activity in Primary Schools (min/wk) ^16^ | 85  (60-120) | 52  (45-60) | 90 | 125  (45-150) | 77  (27-120) | 120 | 119  (37-200) |
| Active Transport in Children aged 10-14 yrs (km/yr)^17^  Walking  Bicycling | 275  424 | N/A | 180  2200 | N/A | 182  26 | 396  79 | 123 N/A |

Note.

^1^World Bank. *GINI Index (World Bank estimate)*. [cited 2021 22 January]; Available from: <https://data.worldbank.org/indicator/SI.POV.GINI>.

^2^Centre d’étude sur la pauvreté et l’exclusion. Poverty, inequality and social exclusion in québec: 2018 Progress Report. <https://www.mtess.gouv.qc.ca/publications/pdf/CEPE_Etat-situation2018_EN.pdf>

^3^OECD Employment: [Length of maternity leave, parental leave, and paid father-specific leave](https://stats.oecd.org/index.aspx?queryid=54760) <https://stats.oecd.org/index.aspx?queryid=54760>

^4^Statistique Canada, L’emploi et le revenu en perspective. <https://www150.statcan.gc.ca/n1/pub/75-001-x/00303/6490-fra.html>

^5^Gouvernement du Québec : Brochure, Régime Québécois d’Assurance Parentale (RQAP) . Quebec, 2017. <https://www.rqap.gouv.qc.ca/sites/default/files/documents/publications/RQAP_Brochure_fr.pdf>; a) Includes 11∙3-12∙6 weeks exclusively entitled to the mother and 18∙7 weeks sharable with the father; b) Includes 2∙3-3∙5 exclusively entitled to the father and 18∙7 weeks sharable with the mother.

^6^Data from UNICEF, Y.Chzhen, A Gromada, G Rees. Are the world´s richest countries family friendly? Policy in the OECD and EU, 2019, UNICEF, Florence.

^7^Statistics Canada, Catalogue No 98-200-X2016012 (2017): Children living in low-income households. Census of population 2016. <https://www12.statcan.gc.ca/census-recensement/2016/as-sa/98-200-x/2016012/98-200-x2016012-eng.pdf>

^8^Data from Statistics Canada CANSIM 282-0211. Labour force characteristics by family age composition, annual. <https://www150.statcan.gc.ca/t1/tbl1/en/cv.action?pid=1410012001>

^9^OECD Net childcare cost for parents using childcare. <https://stats.oecd.org/Index.aspx?DataSetCode=NCC>

^10^Average household income 49,998 Canadian dollars <https://www12.statcan.gc.ca/english/census01/products/standard/prprofile/prprofile.cfm?G=24> Cost of preschool 5 dollars per child per day. Maximum cost: 2 600 dollars

^11^The Joint Research Centre (JRC). [School food policy country factsheets | EU Science Hub (europa.eu)](https://ec.europa.eu/jrc/en/publication/school-food-policy-country-factsheets)

^12^[National School Lunch Program | USDA-FNS](https://www.fns.usda.gov/nslp)

^13^[Lag (1991:2027) om kabelsändningar till allmänheten](https://www.riksdagen.se/sv/dokument-lagar/dokument/svensk-forfattningssamling/lag-19912027-om-kabelsandningar-till_sfs-1991-2027). Sveriges riksdag. 19 december 1991.

^14^Legis Quebec Paragraph [248.](javascript:displayOtherLang(%22se:248%22);) “Subject to what is provided in the regulations, no person may make use of commercial advertising directed at persons under thirteen years of age.” [p-40∙1 - Consumer Protection Act (gouv.qc.ca)](http://legisquebec.gouv.qc.ca/en/showdoc/cs/P-40.1)

^15^Adams J, Hennessy-Priest K, Ingimarsdóttir S, Sheeshka J, Ostbye T, White M. Food advertising during children’s television in Canada and the UK. Arch Dis Child. 2009 ; 94 (9): 658–62 .

^16^UNESCO-NWCPEA, World-wide Survey of School Physical Education – Final Report 2013. Appendix II

^17^Garrard, J.; Active transport: children and young people: an overview of recent evidence. The Victorian Health Promotion Foundation (VicHealth) Dec 2009. Australian numbers are based on data only from Melbourne.

**Supplementary Table S3: Overweight/Obese and Obese Prevalence by Maternal Educational and Household Income. (Weighted Estimates)**

|  | **Overweight/Obese** | | | | | | | |
| --- | --- | --- | --- | --- | --- | --- | --- | --- |
|  | Complete Case Only^a^ | | Maternal Education^b^ (%) | | | Household Income^c^ (%) | | |
|  | n | % | Low | Middle | High | Low | Middle | High |
| ABIS | 487 | 15.2 | 14.3 | 16.2 | 13.8 | 17.4 | 14.5 | 15.4 |
| QLSCD | 283 | 25.9 | 27.5 | 28.6 | 21.7 | 29.5 | 26.5 | 22.0 |
| GenR | 727 | 16.7 | 31.4 | 20.8 | 9.8 | 28.8 | 16.1 | 7.5 |
| NLSCY | 361 | 26.6 | 41.8 | 32.8 | 23.7 | 35.0 | 33.6 | 22.9 |
| LSAC-B | 926 | 24.7 | 29.2 | 27.5 | 17.8 | 31.0 | 25.4 | 16.7 |
| MCS | 3387 | 27.4 | 30.3 | 28.5 | 22.5 | 30.4 | 28.5 | 18.5 |
| USNLSY | 957 | 37.6 | 44.9 | 37.8 | 27.6 | 41.0 | 35.5 | 27.4 |
|  | **Obese** | | | | | | | |
|  | Complete Case Only | | Maternal Education (%) | | | Household Income (%) | | |
|  | n | % | Low | Middle | High | Low | Middle | High |
| ABIS | 83 | 2.6 | 4.4 | 3.1 | 1.2 | 3.4 | 2.3 | 2.8 |
| QLSCD | 71 | 6.5 | 9.9 | 6.9 | 2.6 | 10.9 | 5.9 | 3.8 |
| GenR | 135 | 3.1 | 9.4 | 3.8 | 0.9 | 7.1 | 2.7 | 0.5 |
| NLSCY | 99 | 7.3 | 12.2 | 9.8 | 6.8 | 14.9 | 9.7 | 5.0 |
| LSAC-B | 265 | 7.3 | 11.5 | 8.1 | 4.0 | 8.9 | 8.0 | 3.9 |
| MCS | 804 | 6.5 | 9.1 | 6.5 | 3.9 | 8.6 | 6.6 | 3.0 |
| USNLSY | 401 | 15.8 | 17.9 | 16.1 | 8.6 | 16.2 | 14.0 | 8.3 |

Note. ^a^Complete case only is cohort subsample that excludes missing data to yield valid percentages; see Table 1. ^b^Maternal education harmonized across cohorts into 3 categories based on International Standard Classification of Education: high (ISCED V-VII), middle (ISCED III-IV), low (ISCED I-II). ^c^Household income grouped into high (5^th^ quintile, richest), middle (2^nd^ to 4^th^ quintile), low (1^st^ quintile, poorest).

**Supplementary Table S4: Bivariate Analysis of Risk Ratios for Overweight/Obese at Follow-Up**^a^ **by Maternal Education, Household Income, and Baseline Confounders. (Weighted Estimates)**

|  |  | **ABIS**  Sweden | **QLSCD**  Quebec | **GenR**  Netherlands | **NLSCY**  Canada | **LSAC-B**  Australia | **MCS**  United Kingdom | **USNSLY**  USA |
| --- | --- | --- | --- | --- | --- | --- | --- | --- |
|  | | Relative Risk, RR (95% Confidence Interval) | | | | | | |
| Maternal Education^b^ | |  |  |  |  |  |  |  |
|  | High (Reference) | 1.00 | 1.00 | 1.00 | 1.00 | 1.00 | 1.00 | 1.00 |
|  | Middle | 1.17 (0.99, 1.40) | 1.32 (1.01, 1.72) | 1.96 (1.69, 2.29) | 1.42 (1.04, 1.94) | 1.54 (1.34, 1.77) | 1.37 (1.25, 1.50) | 1.37 (1.18, 1.59) |
|  | Low | 1.04 (0.66, 1.63) | 1.27 (0.93, 1.72) | 2.76 (2.30, 3.31) | 1.85 (1.30, 2.64) | 1.64 (1.35, 1.99) | 1.49 (1.36, 1.65) | 1.63 (1.36, 1.95) |
| Household Income^c^ | |  |  |  |  |  |  |  |
|  | High (Reference) | 1.00 | 1.00 | 1.00 | 1.00 | 1.00 | 1.00 | 1.00 |
|  | Middle | 0.94 (0.77, 1.15) | 1.21 (0.90, 1.63) | 2.11 (1.66, 2.67) | 1.45 (1.03, 2.03) | 1.52 (1.27, 1.82) | 1.76 (1.57, 1.96) | 1.29 (1.07, 1.57) |
|  | Low | 1.13 (0.87, 1.48) | 1.34 (0.91, 1.98) | 3.58 (2.81, 4.57) | 1.57 (0.94, 2.63) | 1.86 (1.52, 2.27) | 1.92 (1.70, 2.16) | 1.49 (1.21, 1.84) |
| Child Sex | |  |  |  |  |  |  |  |
|  | Female (Reference) | 1.00 | 1.00 | 1.00 | 1.00 | 1.00 | 1.00 | 1.00 |
|  | Male | 0.89 (0.75, 1.06) | 1.08 (0.86, 1.35) | 1.29 (1.12, 1.48) | 1.19 (0.9, 1.57) | 0.95 (0.84, 1.07) | 1.23 (1.15, 1.32) | 0.97 (0.86, 1.10) |
| Mother Ethnicity | |  |  |  |  |  |  |  |
|  | Majority / Born in country (Ref) | 1.00 | 1.00 | 1.00 | 1.00 | 1.00 | 1.00 | 1.00 |
|  | Minority / Born outside country | 0.97 (0.66, 1.42) | 1.29 (0.84, 1.98) | 2.24 (1.95, 2.57) | 0.86 (0.51, 1.47) | 0.98 (0.86, 1.11) | 1.30 (1.19, 1.42) | 1.33 (1.20, 1.48) |
| Maternal Age at Child Birth^d^ | | 1.00 (0.98, 1.02) | 0.96 (0.94, 0.97) | 1.01 (0.98, 1.03) | 1.01 (0.97, 1.05) | N/A | 0.99 (0.98, 1.01) | 1.01 (0.99, 1.03) |

Note. ^a^Follow-up age varied by cohort: age 8-9 yrs in ABIS, LSAC; age 8-10 yrs in USNLSY; age 9-10 yrs in GenR; age 10-11 yrs in QLSCD, NLSCY, MCS. ^b^Maternal education harmonized across cohorts into 3 categories based on International Standard Classification of Education: high (ISCED V-VII), middle (ISCED III-IV), low (ISCED I-II). ^c^Household income grouped into high (5^th^ quintile, richest), middle (2^nd^ to 4^th^ quintile), low (1^st^ quintile, poorest). ^d^Maternal age at child birth was not available as a continuous variable for NLSCY; missing data reported for ABIS (n=37, 0.9%), GenR (n=1, 0.0%), LSAC (n=3, 0.0%), USNLSY (n=3, 0.0%).

**Supplementary Table S5: Bivariate Analysis of Risk Ratios for Obese at Follow-Up**^a^ **by Maternal Education, Household Income, and Baseline Confounders. (Weighted Estimates)**

|  | | **ABIS** | **QLSCD** | **GenR** | **NLSCY** | **LSAC-B** | **MCS** | **USNLSY** |
| --- | --- | --- | --- | --- | --- | --- | --- | --- |
|  |  | Sweden | Quebec | Netherlands | Canada | Australia | United Kingdom | USA |
|  | | Relative Risk, RR (95% Confidence Interval) | | | | | | |
| Maternal Education^b^ | |  |  |  |  |  |  |  |
|  | High (Reference) | 1.00 | 1.00 | 1.00 | 1.00 | 1.00 | 1.00 | 1.00 |
|  | Middle | 2.48 (1.42, 4.32) | 2.68 (1.31, 5.47) | 3.47 (2.28, 5.27) | 1.69 (0.91, 3.14) | 2.05 (1.48, 2.83) | 1.71 (1.42, 2.05) | 1.88 (1.42, 2.49) |
|  | Low | 3.56 (1.37, 9.25) | 3.80 (1.88, 7.70) | 6.37 (4.03, 10.07) | 2.08 (1.01, 4.28) | 2.91 (1.98, 4.28) | 2.44(2.03, 2.94) | 2.09 (1.46, 2.98) |
| Household Income^c^ | |  |  |  |  |  |  |  |
|  | High (Reference) | 1.00 | 1.00 | 1.00 | 1.00 | 1.00 | 1.00 | 1.00 |
|  | Middle | 0.81 (0.48, 1.38) | 1.55 (0.74, 3.25) | 7.09 (2.59, 19.15) | 3.50 (1.68, 7.29) | 2.08 (1.44, 3.00) | 2.32 (1.82, 2.95) | 1.69 (1.14, 2.51) |
|  | Low | 1.21 (0.60, 2.42) | 2.85 (1.30, 6.25) | 16.56 (6.04, 45.41) | 4.82 (1.64, 4.15) | 2.30 (1.47, 3.60) | 3.10 (2.40, 4.00) | 1.96 (1.28, 2.99) |
| Child Sex | |  |  |  |  |  |  |  |
|  | Female | 1.00 | 1.00 | 1.00 | 1.00 | 1.00 | 1.00 | 1.00 |
|  | Male | 0.81 (0.51, 1.29) | 1.31 (0.78, 2.20) | 1.49 (1.05, 2.12) | 1.19 (0.9, 1.57) | 1.04 (0.82, 1.31) | 1.06 (0.94, 1.18) | 1.21 (0.97, 1.51) |
| Mother Ethnicity | |  |  |  |  |  |  |  |
|  | Majority / Born in country (Ref) | 1.00 | 1.00 | 1.00 | 1.00 | 1.00 | 1.00 | 1.00 |
|  | Minority / Born outside country | 2.10 (1.01, 4.37) | 0.87 (0.18, 4.32) | 4.23 (2.89, 6.19) | 0.86 (0.51, 1.47) | 0.97 (0.75, 1.25) | 1.39 (1.19, 1.62) | 1.71 (1.41, 2.07) |
| Maternal Age at Child Birth^d^ | | 1.00 (0.95, 1.06) | 0.93 (0.90, 0.97) | 0.98 (0.94, 1.02) | 1.01 (0.97, 1.05) | N/A | 1.00 (0.97, 1.03) | 1.01 (0.98, 1.05) |

Note. ^a^Follow-up age varied by cohort: age 8-9 yrs in ABIS, LSAC; age 8-10 yrs in USNLSY; age 9-10 yrs in GenR; age 10-11 yrs in QLSCD, NLSCY, MCS. ^b^Maternal education harmonized across cohorts into 3 categories based on International Standard Classification of Education: high (ISCED V-VII), middle (ISCED III-IV), low (ISCED I-II). ^c^Household income grouped into high (5^th^ quintile, richest), middle (2^nd^ to 4^th^ quintile), low (1^st^ quintile, poorest). ^d^Maternal age at child birth was not available as a continuous variable for NLSCY; missing data reported for ABIS (n=37, 0.9%), GenR (n=1, 0.0%), LSAC (n=3, 0.0%), USNLSY (n=3, 0.0%).

|  |  | **ABIS**  Sweden | **QLSCD**  Quebec | **GenR**  Netherlands | **NLSCY**  Canada | **LSAC-B**  Australia | **MCS**  UK | **USNLSY**  USA |
| --- | --- | --- | --- | --- | --- | --- | --- | --- |
|  |  | Risk Ratio, (95% Confidence Interval) | | | | | | |
|  |  | **Overweight/Obese**^a^ | | | | | | |
| Maternal Education^b^ | | | | | | | | |
|  | High (Reference) | 1.00 | 1.00 | 1.00 | 1.00 | 1.00 | 1.00 | 1.00 |
|  | Middle | 1.20 (1.00, 1.43) | 1.28 (0.96, 1.72) | 1.58 (1.33, 1.88) | 1.32 (0.98, 1.77) | 1.41 (1.23, 1.62) | 1.18 (1.06, 1.32) | 1.37 (1.16, 1.63) |
|  | Low | 1.00 (0.63, 1.57) | 1.22 (0.87, 1.71) | 2.05 (1.68, 2.49) | 1.56 (1.07, 2.27) | 1.41 (1.15, 1.74) | 1.18 (1.08, 1.29) | 1.60 (1.27, 2.01) |
| Household Income^c^ | | | | | | | | |
|  | High (Reference) | 1.00 | 1.00 | 1.00 | 1.00 | 1.00 | 1.00 | 1.00 |
|  | Middle | 0.94 (0.76, 1.16) | 1.13 (0.80, 1.59) | 1.66 (1.31, 2.11) | 1.49 (1.06, 2.08) | 1.41 (1.17, 1.68) | 1.48 (1.31, 1.65) | 1.17 (0.96, 1.43) |
|  | Low | 1.13 (0.85, 1.50) | 1.21 (0.76, 1.93) | 1.96 (1.50, 2.57) | 1.60 (0.95, 2.70) | 1.64 (1.33, 2.04) | 1.53 (1.33, 1.77) | 1.14 (0.90, 1.45) |
|  |  | **Obese**^a^ | | | | | | |
| Maternal Education^b^ | | | | | | | | |
|  | High (Reference) | 1.00 | 1.00 | 1.00 | 1.00 | 1.00 | 1.00 | 1.00 |
|  | Middle | 2.57 (1.46, 4.55) | 2.52 (1.28, 4.94) | 2.54 (1.48, 4.35) | 1.21 (0.63, 2.35) | 1.90 (1.36, 2.66) | 1.50 (1.19, 1.90) | 1.86 (1.33, 2.60) |
|  | Low | 3.65 (1.38, 9.69) | 3.34 (1.56, 7.13) | 5.01 (2.85, 8.80) | 1.23 (0.54, 2.76) | 2.65 (1.77, 3.96) | 1.86 (1.42, 2.45) | 1.98 (1.26, 3.12) |
| Household Income^c^ | | | | | | | | |
|  | High (Reference) | 1.00 | 1.00 | 1.00 | 1.00 | 1.00 | 1.00 | 1.00 |
|  | Middle | 0.65 (0.38, 1.70) | 1.06 (0.50, 2.24) | 3.97 (1.44, 10.94) | 1.93 (0.75, 4.98) | 1.75 (1.20, 2.55) | 1.94 (1.40, 2.68) | 1.39 (0.93, 2.08) |
|  | Low | 0.79 (0.37, 1.13) | 1.69 (0.62, 4.59) | 4.66 (1.63, 13.33) | 2.97 (0.95, 9.25) | 1.78 (1.11, 2.88) | 2.30 (1.58, 3.34) | 1.24 (0.77, 1.99) |

**Table S6.** Risk Ratios for Overweight/Obese and Obese at Follow-Up with both SES measures simultaneously included (Household Income and Maternal Education at Baseline) using Adjusted Multivariate Regression. (Weighted estimates)

Note. ^a^Adjusted for child sex, mother ethnicity, maternal age at child birth, maternal education, and household income. Maternal education and household income exposures at baseline. Overweight/Obese and Obese outcomes at follow-up; age varied by cohort: age 8-9 yrs in ABIS, LSAC; age 8-10 yrs in USNLSY; age 9-10 yrs in GenR; age 10-11 yrs in QLSCD, NLSCY, MCS. ^b^Maternal education harmonized across cohorts into 3 categories based on International Standard Classification of Education: high (ISCED V-VII), middle (ISCED III-IV), low (ISCED I-II). ^c^Household income grouped into high (5^th^ quintile, richest), middle (2^nd^ to 4^th^ quintile), low (1^st^ quintile, poorest).
