# Supplementary figures and images for: Household income and maternal education in early childhood and risk of overweight and obesity in late childhood: Findings from seven birth cohort studies in six high-income countries
Source: Int J Obes (Lond). 2022 Jul 11;46(9):1703–11. doi: 10.1038/s41366-022-01171-7 (PMC9395266; doi:10.1038/s41366-022-01171-7)

**Supplementary Figure 1: Correlation of Slope index of inequality and GINI coeffient**


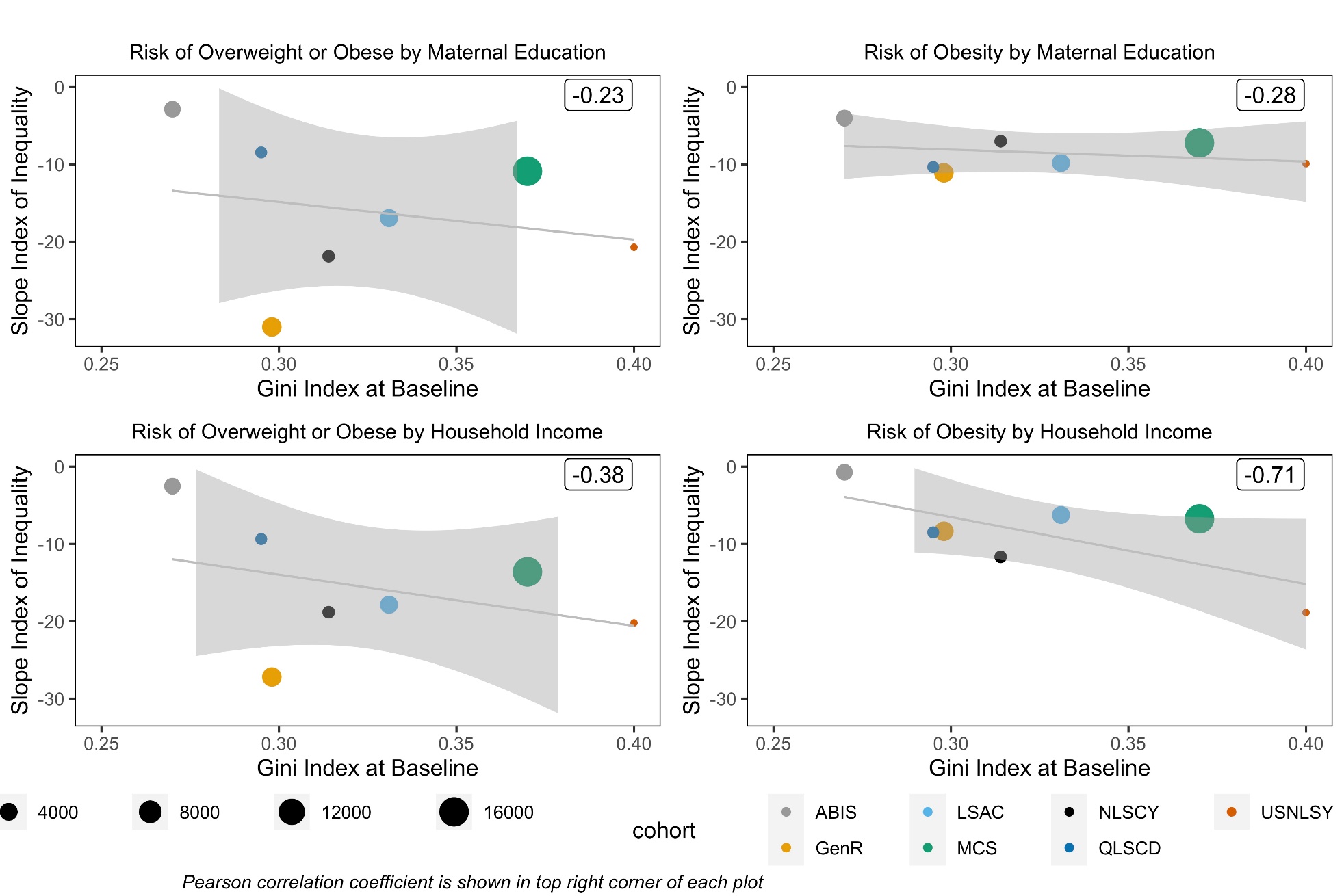

Supplement: Supplementary file 3 — EPOCH Obesity Supplementary Figure 1 SII and GINI [file 41366_2022_1171_MOESM3_ESM.docx]
